# Supplementary material for: Identification of photoperiod-induced specific miRNAs in the adrenal glands of Sunite sheep (Ovis aries)
Source: Front Vet Sci. 2022 Jul 22;9:888207. doi: 10.3389/fvets.2022.888207 (PMC9354845; doi:10.3389/fvets.2022.888207)
Supplement: Supplementary file 1 [file Data_Sheet_1.ZIP › Supplementary file/miRDeep2 file.zip!/miRDeep2 file/Novel_Structure/SP42A2/Novel_534.pdf]

Provisional ID	:	NC_019477.2_35131
Score total	:	6.3
Score for star read(s)	:	3.9
Score for read counts	:	-0.8
Score for mfe	:	1.7
Score for randfold	:	1.6
Score for cons. seed	:	
Total read count	:	10
Mature read count	:	6
Loop read count	:	0
Star read count	:	4

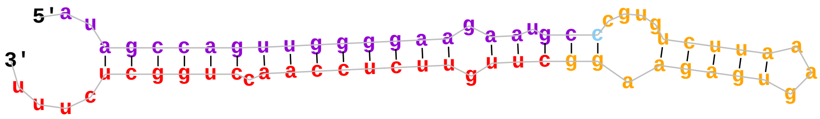

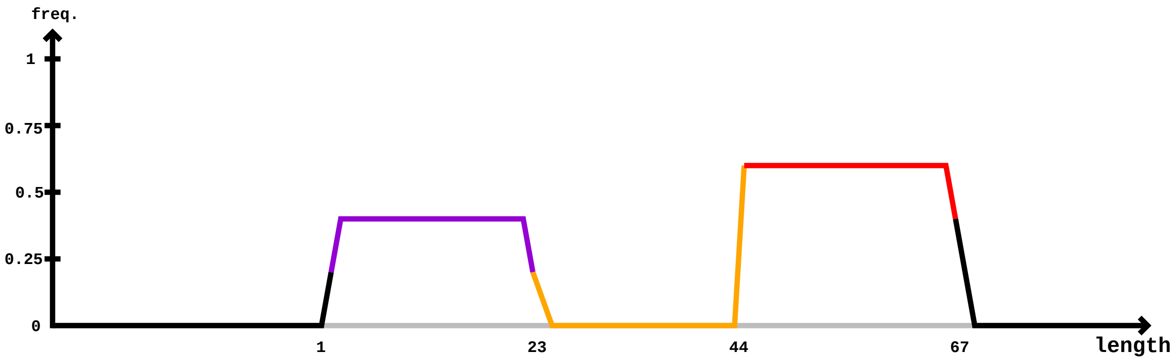


```
                                   Star                                    Mature
5'- ccagggaaaggcuaauccuauugaggcauagccaguuggggaagaaugcccgugucuuaaagugagaaggcuuguucuccaaccuggcucuuuauggcugcuggggaaaggc   -3'    obs
    ccagggaaaggcuaauccuauugaggcauagccaguuggggaagaaugcccgugucuuaaagugagaaggcuuguucuccaaccuggcucuuuauggcugcuggggaaaggc          exp
    .........(((..((((....((.(((((((((((((((((.((.(((....(((((...))))).))))).)))))))))).))))))....))).))....))))..)))   reads     mm       sample
    ...........................auagccaguuggggaagaaug................................................................   1         0        seq
    ...........................auagccaguuggggaagaaugc...............................................................   1         0        seq
    ............................uagccaguuggggaagaaug................................................................   1         0        seq
    ............................uagccaguuggggaagaaugc...............................................................   1         0        seq
    .....................................................................cuuguucuccaaccuggcucuu.....................   2         0        seq
    .....................................................................cuuguucuccaaccuggcucuuu....................   4         0        seq
```
